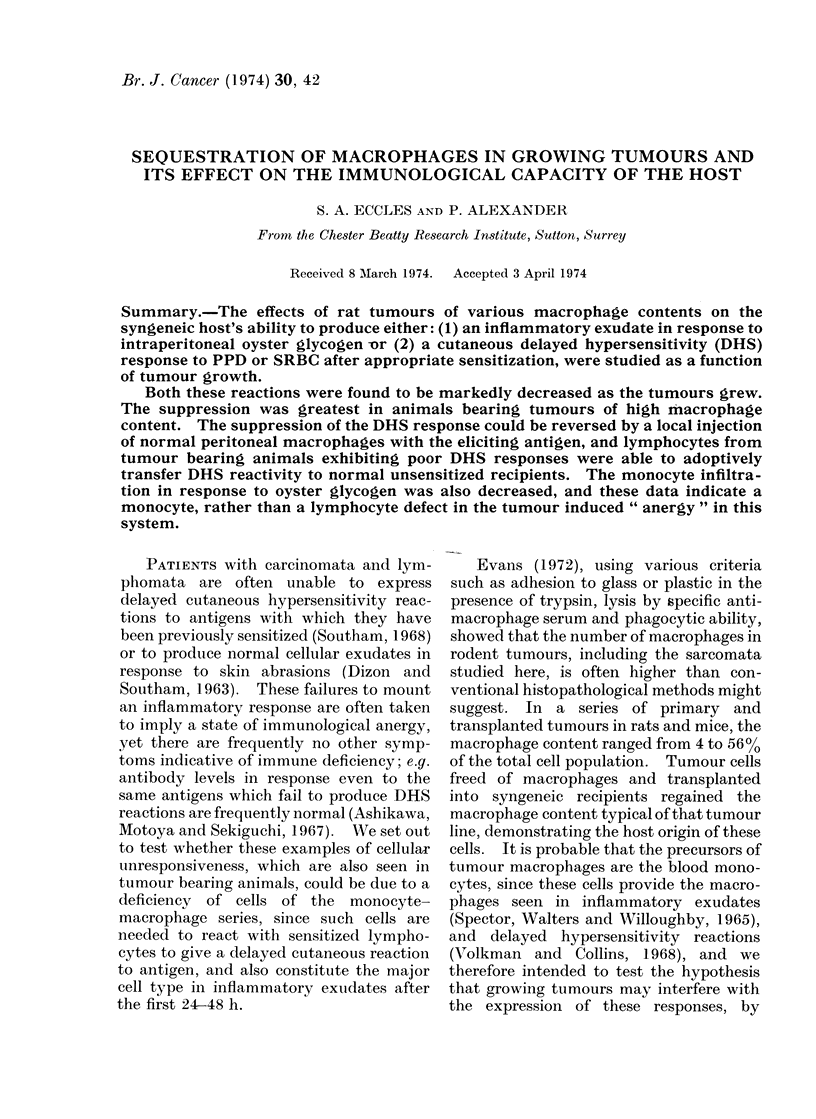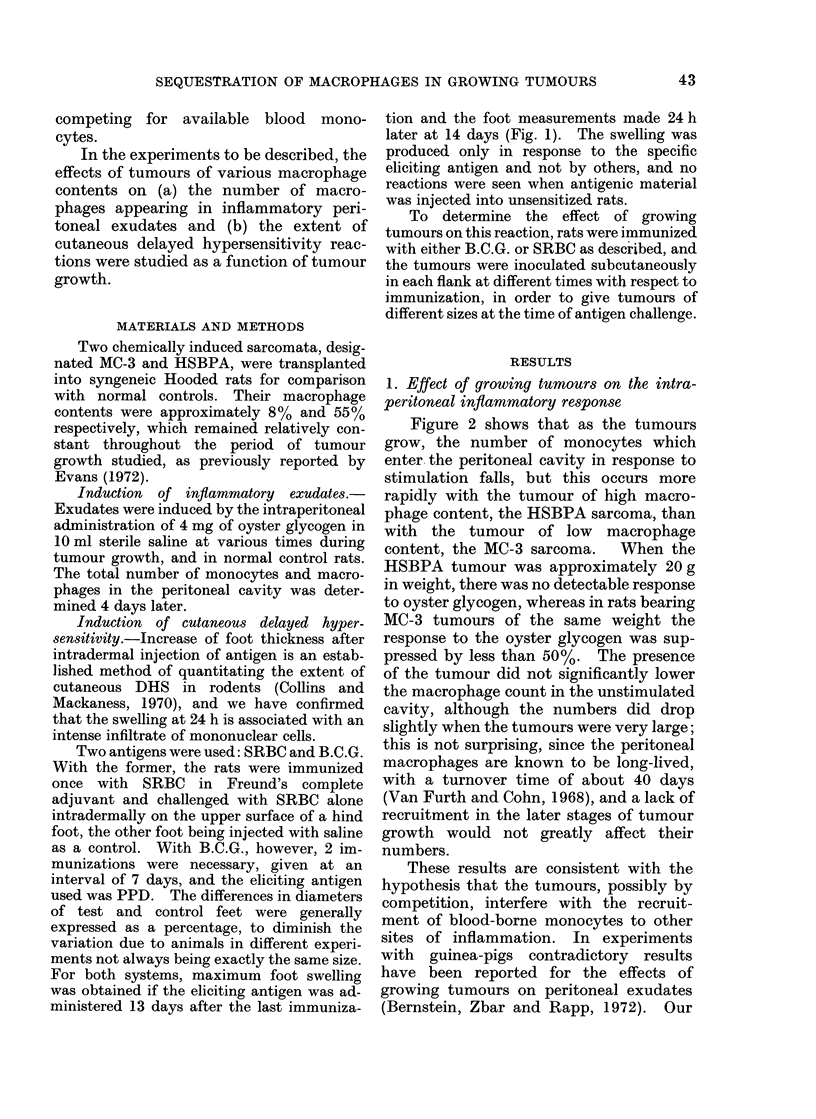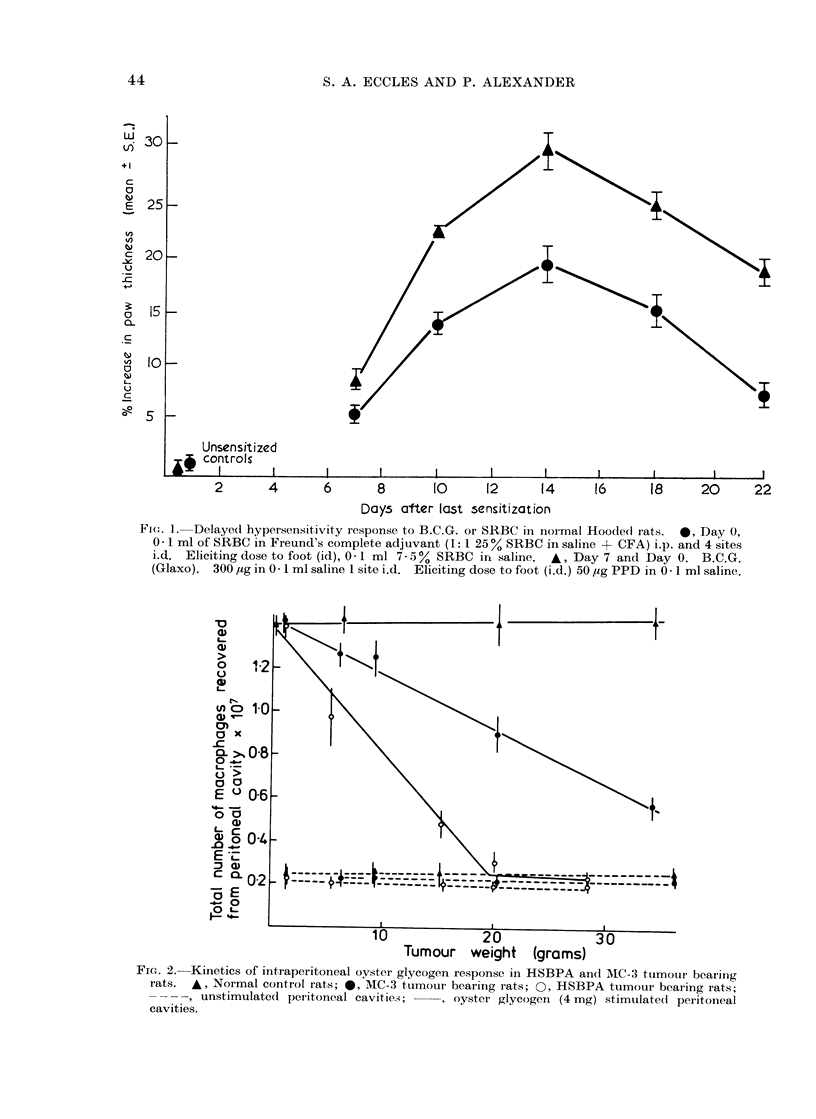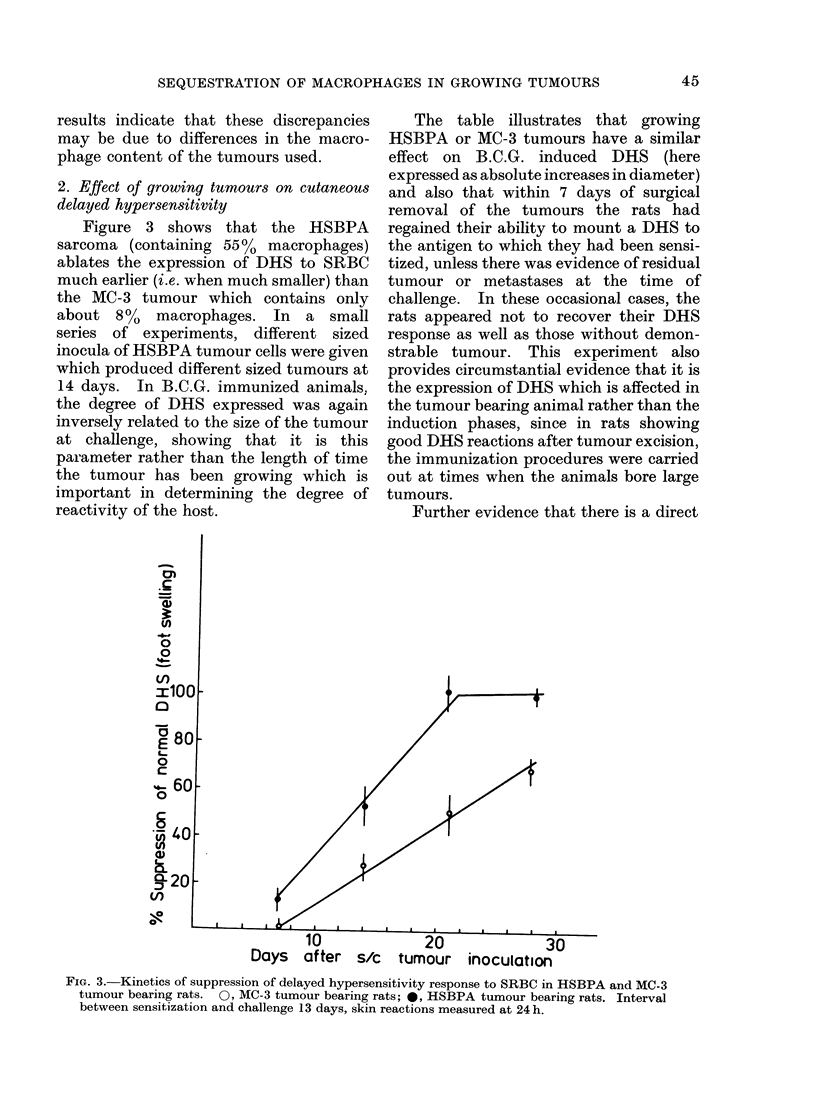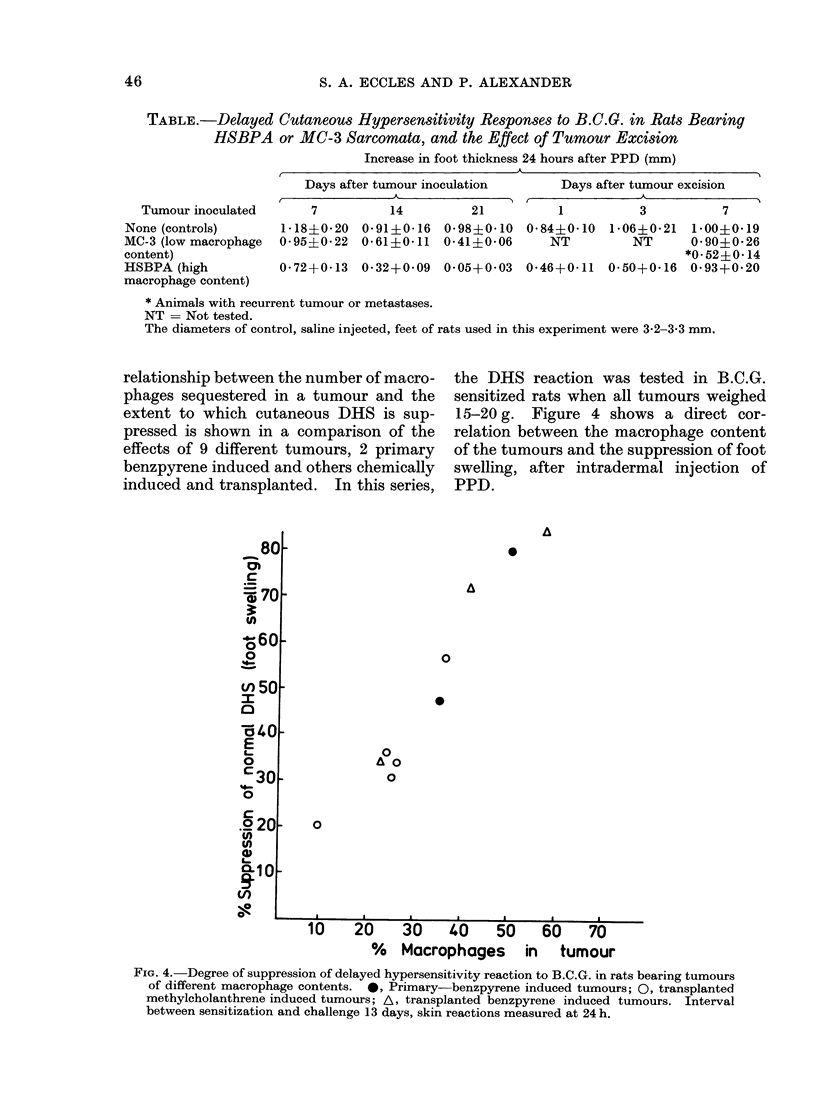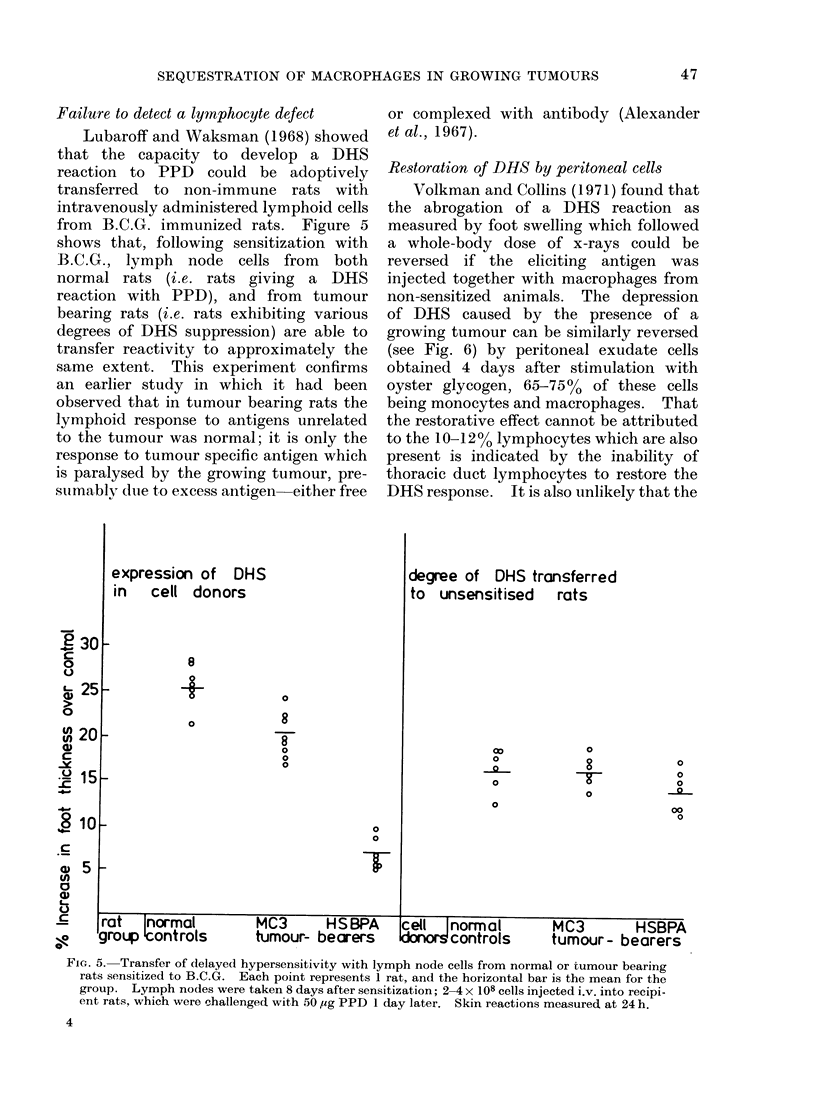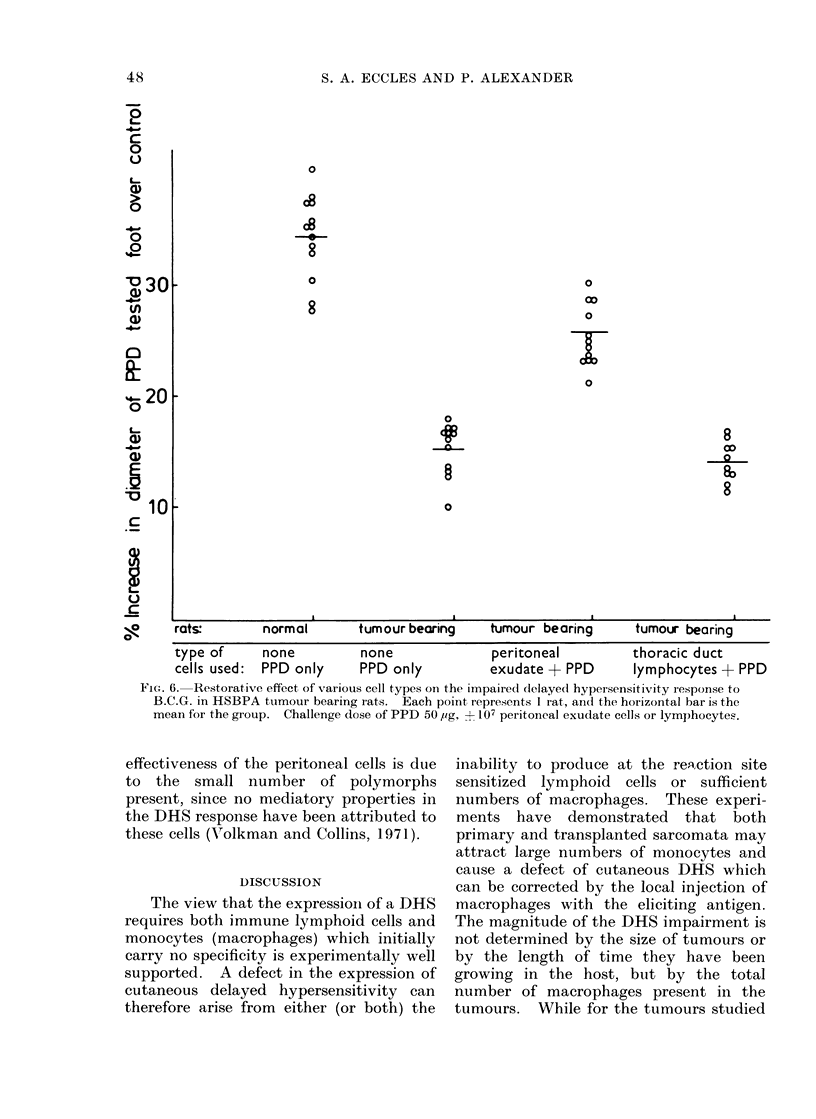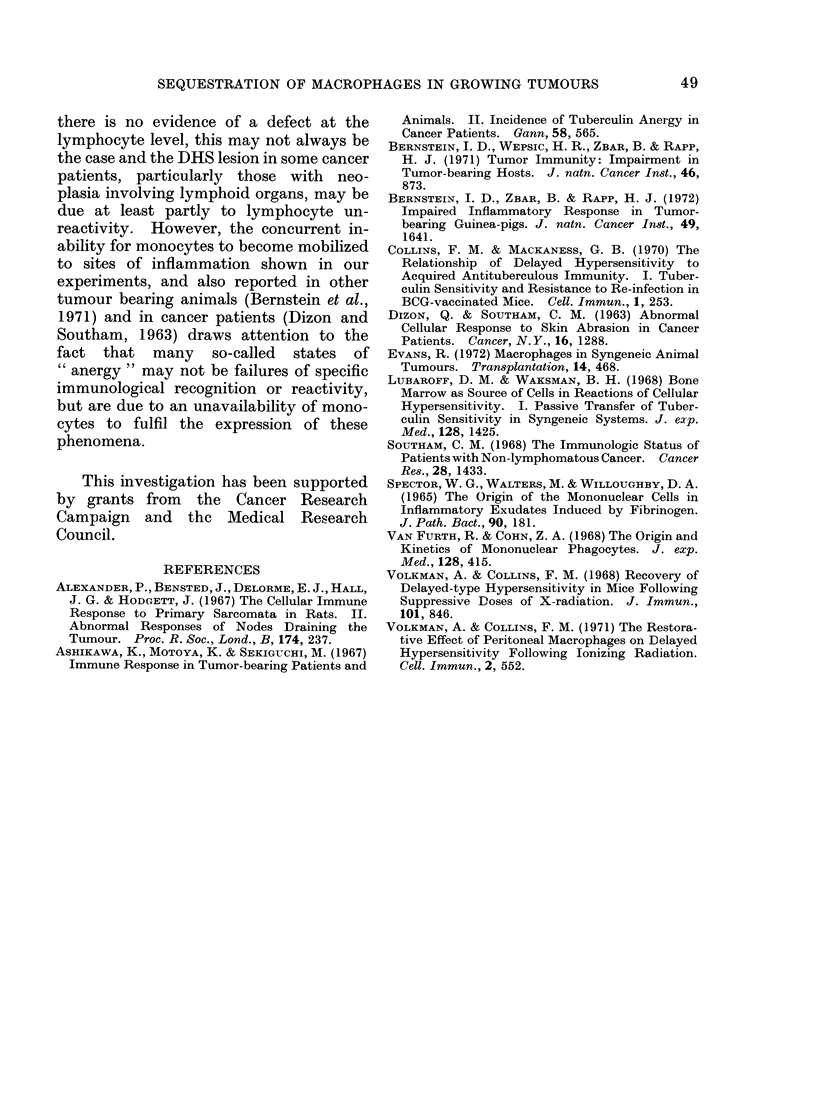# Sequestration of Macrophages in Growing Tumours and its Effect on the Immunological Capacity of the Host

**DOI:** 10.1038/bjc.1974.111

**Published:** 1974-07

**Authors:** S. A. Eccles, P. Alexander

## Abstract

The effects of rat tumours of various macrophage contents on the syngeneic host's ability to produce either: (1) an inflammatory exudate in response to intraperitoneal oyster glycogen or (2) a cutaneous delayed hypersensitivity (DHS) response to PPD or SRBC after appropriate sensitization, were studied as a function of tumour growth.

Both these reactions were found to be markedly decreased as the tumours grew. The suppression was greatest in animals bearing tumours of high macrophage content. The suppression of the DHS response could be reversed by a local injection of normal peritoneal macrophages with the eliciting antigen, and lymphocytes from tumour bearing animals exhibiting poor DHS responses were able to adoptively transfer DHS reactivity to normal unsensitized recipients. The monocyte infiltration in response to oyster glycogen was also decreased, and these data indicate a monocyte, rather than a lymphocyte defect in the tumour induced “anergy” in this system.


*Br. J. Cancer* (1974) **30**, 42

### SEQUESTRATION OF MACROPHAGES IN GROWING TUMOURS AND ITS EFFECT ON THE IMMUNOLOGICAL CAPACITY OF THE HOST

S. A. ECCLES AND P. ALEXANDER

*From the Chester Beatty Research Institute, Sutton, Surrey*

Received 8 March 1974. Accepted 3 April 1974

**Summary.—The effects of rat tumours of various macrophage contents on the syngeneic host's ability to produce either: (1) an inflammatory exudate in response to intraperitoneal oyster glycogen or (2) a cutaneous delayed hypersensitivity (DHS) response to PPD or SRBC after appropriate sensitization, were studied as a function of tumour growth.**

**Both these reactions were found to be markedly decreased as the tumours grew. The suppression was greatest in animals bearing tumours of high macrophage content. The suppression of the DHS response could be reversed by a local injection of normal peritoneal macrophages with the eliciting antigen, and lymphocytes from tumour bearing animals exhibiting poor DHS responses were able to adoptively transfer DHS reactivity to normal unsensitized recipients. The monocyte infiltration in response to oyster glycogen was also decreased, and these data indicate a monocyte, rather than a lymphocyte defect in the tumour induced " anergy " in this system.**

PATIENTS with carcinomata and lymphomata are often unable to express delayed cutaneous hypersensitivity reactions to antigens with which they have been previously sensitized (Southam, 1968) or to produce normal cellular exudates in response to skin abrasions (Dizon and Southam, 1963). These failures to mount an inflammatory response are often taken to imply a state of immunological anergy, yet there are frequently no other symptoms indicative of immune deficiency; *e.g.* antibody levels in response even to the same antigens which fail to produce DHS reactions are frequently normal (Ashikawa, Motoya and Sekiguchi, 1967). We set out to test whether these examples of cellular unresponsiveness, which are also seen in tumour bearing animals, could be due to a deficiency of cells of the monocyte–macrophage series, since such cells are needed to react with sensitized lymphocytes to give a delayed cutaneous reaction to antigen, and also constitute the major cell type in inflammatory exudates after the first 24–48 h.

Evans (1972), using various criteria such as adhesion to glass or plastic in the presence of trypsin, lysis by specific anti-macrophage serum and phagocytic ability, showed that the number of macrophages in rodent tumours, including the sarcomata studied here, is often higher than conventional histopathological methods might suggest. In a series of primary and transplanted tumours in rats and mice, the macrophage content ranged from 4 to 56% of the total cell population. Tumour cells freed of macrophages and transplanted into syngeneic recipients regained the macrophage content typical of that tumour line, demonstrating the host origin of these cells. It is probable that the precursors of tumour macrophages are the blood monocytes, since these cells provide the macrophages seen in inflammatory exudates (Spector, Walters and Willoughby, 1965), and delayed hypersensitivity reactions (Volkman and Collins, 1968), and we therefore intended to test the hypothesis that growing tumours may interfere with the expression of these responses, by competing for available blood monocytes.

In the experiments to be described, the effects of tumours of various macrophage contents on (a) the number of macrophages appearing in inflammatory peritoneal exudates and (b) the extent of cutaneous delayed hypersensitivity reactions were studied as a function of tumour growth.

### MATERIALS AND METHODS

Two chemically induced sarcomata, designated MC-3 and HSBPA, were transplanted into syngeneic Hooded rats for comparison with normal controls. Their macrophage contents were approximately 8% and 55% respectively, which remained relatively constant throughout the period of tumour growth studied, as previously reported by Evans (1972).

*Induction of inflammatory exudates.*—Exudates were induced by the intraperitoneal administration of 4 mg of oyster glycogen in 10 ml sterile saline at various times during tumour growth, and in normal control rats. The total number of monocytes and macrophages in the peritoneal cavity was determined 4 days later.

*Induction of cutaneous delayed hypersensitivity.*—Increase of foot thickness after intradermal injection of antigen is an established method of quantitating the extent of cutaneous DHS in rodents (Collins and Mackaness, 1970), and we have confirmed that the swelling at 24 h is associated with an intense infiltrate of mononuclear cells.

Two antigens were used: SRBC and B.C.G. With the former, the rats were immunized once with SRBC in Freund's complete adjuvant and challenged with SRBC alone intradermally on the upper surface of a hind foot, the other foot being injected with saline as a control. With B.C.G., however, 2 immunizations were necessary, given at an interval of 7 days, and the eliciting antigen used was PPD. The differences in diameters of test and control feet were generally expressed as a percentage, to diminish the variation due to animals in different experiments not always being exactly the same size. For both systems, maximum foot swelling was obtained if the eliciting antigen was administered 13 days after the last immunization and the foot measurements made 24 h later at 14 days (Fig. 1). The swelling was produced only in response to the specific eliciting antigen and not by others, and no reactions were seen when antigenic material was injected into unsensitized rats.

To determine the effect of growing tumours on this reaction, rats were immunized with either B.C.G. or SRBC as described, and the tumours were inoculated subcutaneously in each flank at different times with respect to immunization, in order to give tumours of different sizes at the time of antigen challenge.

### RESULTS

### 1. *Effect of growing tumours on the intraperitoneal inflammatory response*

Figure 2 shows that as the tumours grow, the number of monocytes which enter the peritoneal cavity in response to stimulation falls, but this occurs more rapidly with the tumour of high macrophage content, the HSBPA sarcoma, than with the tumour of low macrophage content, the MC-3 sarcoma. When the HSBPA tumour was approximately 20 g in weight, there was no detectable response to oyster glycogen, whereas in rats bearing MC-3 tumours of the same weight the response to the oyster glycogen was suppressed by less than 50%. The presence of the tumour did not significantly lower the macrophage count in the unstimulated cavity, although the numbers did drop slightly when the tumours were very large; this is not surprising, since the peritoneal macrophages are known to be long-lived, with a turnover time of about 40 days (Van Furth and Cohn, 1968), and a lack of recruitment in the later stages of tumour growth would not greatly affect their numbers.

These results are consistent with the hypothesis that the tumours, possibly by competition, interfere with the recruitment of blood-borne monocytes to other sites of inflammation. In experiments with guinea-pigs contradictory results have been reported for the effects of growing tumours on peritoneal exudates (Bernstein, Zbar and Rapp, 1972). Our

FIG. 1.—Delayed hypersensitivity response to B.C.G. or SRBC in normal Hooded rats. ●, Day 0, 0·1 ml of SRBC in Freund's complete adjuvant (1: 1 25% SRBC in saline + CFA) i.p. and 4 sites i.d. Eliciting dose to foot (id), 0·1 ml 7·5% SRBC in saline. ▲, Day 7 and Day 0. B.C.G. (Glaxo). 300 μg in 0·1 ml saline 1 site i.d. Eliciting dose to foot (i.d.) 50 μg PPD in 0·1 ml saline.

FIG. 2.—Kinetics of intraperitoneal oyster glycogen response in HSBPA and MC-3 tumour bearing rats. ▲, Normal control rats; ●, MC-3 tumour bearing rats; ○, HSBPA tumour bearing rats; – – – –, unstimulated peritoneal cavities; ——, oyster glycogen (4 mg) stimulated peritoneal cavities.

results indicate that these discrepancies may be due to differences in the macrophage content of the tumours used.

2. *Effect of growing tumours on cutaneous delayed hypersensitivity*

Figure 3 shows that the HSBPA sarcoma (containing 55% macrophages) ablates the expression of DHS to SRBC much earlier (*i.e.* when much smaller) than the MC-3 tumour which contains only about 8% macrophages. In a small series of experiments, different sized inocula of HSBPA tumour cells were given which produced different sized tumours at 14 days. In B.C.G. immunized animals, the degree of DHS expressed was again inversely related to the size of the tumour at challenge, showing that it is this parameter rather than the length of time the tumour has been growing which is important in determining the degree of reactivity of the host.

The table illustrates that growing HSBPA or MC-3 tumours have a similar effect on B.C.G. induced DHS (here expressed as absolute increases in diameter) and also that within 7 days of surgical removal of the tumours the rats had regained their ability to mount a DHS to the antigen to which they had been sensitized, unless there was evidence of residual tumour or metastases at the time of challenge. In these occasional cases, the rats appeared not to recover their DHS response as well as those without demonstrable tumour. This experiment also provides circumstantial evidence that it is the expression of DHS which is affected in the tumour bearing animal rather than the induction phases, since in rats showing good DHS reactions after tumour excision, the immunization procedures were carried out at times when the animals bore large tumours.

Further evidence that there is a direct

FIG. 3.—Kinetics of suppression of delayed hypersensitivity response to SRBC in HSBPA and MC-3 tumour bearing rats. ○, MC-3 tumour bearing rats; ●, HSBPA tumour bearing rats. Interval between sensitization and challenge 13 days, skin reactions measured at 24 h.

TABLE.—*Delayed Cutaneous Hypersensitivity Responses to B.C.G. in Rats Bearing HSBPA or MC-3 Sarcomata, and the Effect of Tumour Excision*

| | Increase in foot thickness 24 hours after PPD (mm) | | | | | |
|---|---|---|---|---|---|---|
| | Days after tumour inoculation | | | Days after tumour excision | | |
| Tumour inoculated | 7 | 14 | 21 | 1 | 3 | 7 |
| None (controls) | 1·18±0·20 | 0·91±0·16 | 0·98±0·10 | 0·84±0·10 | 1·06±0·21 | 1·00±0·19 |
| MC-3 (low macrophage content) | 0·95±0·22 | 0·61±0·11 | 0·41±0·06 | NT | NT | 0·90±0·26<br>*0·52±0·14 |
| HSBPA (high macrophage content) | 0·72+0·13 | 0·32+0·09 | 0·05+0·03 | 0·46+0·11 | 0·50+0·16 | 0·93+0·20 |

* Animals with recurrent tumour or metastases.
NT = Not tested.
The diameters of control, saline injected, feet of rats used in this experiment were 3·2–3·3 mm.

relationship between the number of macrophages sequestered in a tumour and the extent to which cutaneous DHS is suppressed is shown in a comparison of the effects of 9 different tumours, 2 primary benzpyrene induced and others chemically induced and transplanted. In this series, the DHS reaction was tested in B.C.G. sensitized rats when all tumours weighed 15–20 g. Figure 4 shows a direct correlation between the macrophage content of the tumours and the suppression of foot swelling, after intradermal injection of PPD.

FIG. 4.—Degree of suppression of delayed hypersensitivity reaction to B.C.G. in rats bearing tumours of different macrophage contents. ●, Primary—benzpyrene induced tumours; ○, transplanted methylcholanthrene induced tumours; △, transplanted benzpyrene induced tumours. Interval between sensitization and challenge 13 days, skin reactions measured at 24 h.

*Failure to detect a lymphocyte defect*

Lubaroff and Waksman (1968) showed that the capacity to develop a DHS reaction to PPD could be adoptively transferred to non-immune rats with intravenously administered lymphoid cells from B.C.G. immunized rats. Figure 5 shows that, following sensitization with B.C.G., lymph node cells from both normal rats (*i.e.* rats giving a DHS reaction with PPD), and from tumour bearing rats (*i.e.* rats exhibiting various degrees of DHS suppression) are able to transfer reactivity to approximately the same extent. This experiment confirms an earlier study in which it had been observed that in tumour bearing rats the lymphoid response to antigens unrelated to the tumour was normal; it is only the response to tumour specific antigen which is paralysed by the growing tumour, presumably due to excess antigen—either free or complexed with antibody (Alexander *et al.*, 1967).

*Restoration of DHS by peritoneal cells*

Volkman and Collins (1971) found that the abrogation of a DHS reaction as measured by foot swelling which followed a whole-body dose of x-rays could be reversed if the eliciting antigen was injected together with macrophages from non-sensitized animals. The depression of DHS caused by the presence of a growing tumour can be similarly reversed (see Fig. 6) by peritoneal exudate cells obtained 4 days after stimulation with oyster glycogen, 65–75% of these cells being monocytes and macrophages. That the restorative effect cannot be attributed to the 10–12% lymphocytes which are also present is indicated by the inability of thoracic duct lymphocytes to restore the DHS response. It is also unlikely that the

FIG. 5.—Transfer of delayed hypersensitivity with lymph node cells from normal or tumour bearing rats sensitized to B.C.G. Each point represents 1 rat, and the horizontal bar is the mean for the group. Lymph nodes were taken 8 days after sensitization; $2–4 \times 10^8$ cells injected i.v. into recipient rats, which were challenged with 50 $\mu g$ PPD 1 day later. Skin reactions measured at 24 h.

FIG. 6.—Restorative effect of various cell types on the impaired delayed hypersensitivity response to B.C.G. in HSBPA tumour bearing rats. Each point represents 1 rat, and the horizontal bar is the mean for the group. Challenge dose of PPD 50 $\mu$g, $\pm 10^7$ peritoneal exudate cells or lymphocytes.

effectiveness of the peritoneal cells is due to the small number of polymorphs present, since no mediatory properties in the DHS response have been attributed to these cells (Volkman and Collins, 1971).

DISCUSSION

The view that the expression of a DHS requires both immune lymphoid cells and monocytes (macrophages) which initially carry no specificity is experimentally well supported. A defect in the expression of cutaneous delayed hypersensitivity can therefore arise from either (or both) the inability to produce at the reaction site sensitized lymphoid cells or sufficient numbers of macrophages. These experiments have demonstrated that both primary and transplanted sarcomata may attract large numbers of monocytes and cause a defect of cutaneous DHS which can be corrected by the local injection of macrophages with the eliciting antigen. The magnitude of the DHS impairment is not determined by the size of tumours or by the length of time they have been growing in the host, but by the total number of macrophages present in the tumours. While for the tumours studied there is no evidence of a defect at the lymphocyte level, this may not always be the case and the DHS lesion in some cancer patients, particularly those with neoplasia involving lymphoid organs, may be due at least partly to lymphocyte unreactivity. However, the concurrent inability for monocytes to become mobilized to sites of inflammation shown in our experiments, and also reported in other tumour bearing animals (Bernstein *et al.*, 1971) and in cancer patients (Dizon and Southam, 1963) draws attention to the fact that many so-called states of " anergy " may not be failures of specific immunological recognition or reactivity, but are due to an unavailability of monocytes to fulfil the expression of these phenomena.

This investigation has been supported by grants from the Cancer Research Campaign and thc Medical Research Council.

### REFERENCES

ALEXANDER, P., BENSTED, J., DELORME, E. J., HALL, J. G. & HODGETT, J. (1967) The Cellular Immune Response to Primary Sarcomata in Rats. II. Abnormal Responses of Nodes Draining the Tumour. *Proc. R. Soc., Lond., B*, **174**, 237.

ASHIKAWA, K., MOTOYA, K. & SEKIGUCHI, M. (1967) Immune Response in Tumor-bearing Patients and Animals. II. Incidence of Tuberculin Anergy in Cancer Patients. *Gann*, **58**, 565.

BERNSTEIN, I. D., WEPSIC, H. R., ZBAR, B. & RAPP, H. J. (1971) Tumor Immunity: Impairment in Tumor-bearing Hosts. *J. natn. Cancer Inst.*, **46**, 873.

BERNSTEIN, I. D., ZBAR, B. & RAPP, H. J. (1972) Impaired Inflammatory Response in Tumor-bearing Guinea-pigs. *J. natn. Cancer Inst.*, **49**, 1641.

COLLINS, F. M. & MACKANESS, G. B. (1970) The Relationship of Delayed Hypersensitivity to Acquired Antituberculous Immunity. I. Tuberculin Sensitivity and Resistance to Re-infection in BCG-vaccinated Mice. *Cell. Immun.*, **1**, 253.

DIZON, Q. & SOUTHAM, C. M. (1963) Abnormal Cellular Response to Skin Abrasion in Cancer Patients. *Cancer, N.Y.*, **16**, 1288.

EVANS, R. (1972) Macrophages in Syngeneic Animal Tumours. *Transplantation*, **14**, 468.

LUBAROFF, D. M. & WAKSMAN, B. H. (1968) Bone Marrow as Source of Cells in Reactions of Cellular Hypersensitivity. I. Passive Transfer of Tuberculin Sensitivity in Syngeneic Systems. *J. exp. Med.*, **128**, 1425.

SOUTHAM, C. M. (1968) The Immunologic Status of Patients with Non-lymphomatous Cancer. *Cancer Res.*, **28**, 1433.

SPECTOR, W. G., WALTERS, M. & WILLOUGHBY, D. A. (1965) The Origin of the Mononuclear Cells in Inflammatory Exudates Induced by Fibrinogen. *J. Path. Bact.*, **90**, 181.

VAN FURTH, R. & COHN, Z. A. (1968) The Origin and Kinetics of Mononuclear Phagocytes. *J. exp. Med.*, **128**, 415.

VOLKMAN, A. & COLLINS, F. M. (1968) Recovery of Delayed-type Hypersensitivity in Mice Following Suppressive Doses of X-radiation. *J. Immun.*, **101**, 846.

VOLKMAN, A. & COLLINS, F. M. (1971) The Restorative Effect of Peritoneal Macrophages on Delayed Hypersensitivity Following Ionizing Radiation. *Cell. Immun.*, **2**, 552.